# Emotional Intelligence and Behavioural Addictions: A Systematic Review

**DOI:** 10.3390/jcm14041125

**Published:** 2025-02-10

**Authors:** Roberta Biolcati, Zeynep Özal, Federica Ambrosini, Paola Villano, Laura Palareti, Giacomo Mancini

**Affiliations:** Department of Education Sciences “G.M. Bertin”, Alma Mater Studiorum University of Bologna, 40126 Bologna, Italy; r.biolcati@unibo.it (R.B.); zeynep.ozal2@unibo.it (Z.Ö.); federica.ambrosini3@unibo.it (F.A.); paola.villano@unibo.it (P.V.); laura.palareti2@unibo.it (L.P.)

**Keywords:** emotional intelligence, behavioural addictions, internet addiction, social media addiction, internet gaming disorder, eating disorders, compulsive buying, systematic review

## Abstract

**Background:** The role of emotional intelligence on the experience of behavioural addictions is a growing area of research interest. However, there are operationalisation issues in studying both emotional intelligence and behavioural addictions separately. This review aims to report on the existing literature of studies exploring the relationship between these two concepts, and to identify gaps in research practice in order to inform future studies. **Methods:** A search, covering the date range of 2013–2024, conducted in five databases in August 2024 identified 43 articles, reported according to PRISMA 2020 guidelines. The findings are discussed under four subheadings: technology-related behavioural addiction, internet gaming disorder, eating disorders, and consumer behaviour and compulsive buying. **Results:** Overall, the results show that emotional intelligence is negatively correlated with behavioural addictions and plays both a moderating and mediating role in the associations between behavioural addictions and other negative outcomes such as suicidal ideation, rumination, fear of missing out, and depression. **Conclusions:** In addition to summarising studies and controversial discussions on emotional intelligence and behavioural addictions, this review suggests possible roadmaps to ensure more accurate research outcomes by highlighting the importance of theoretical and methodological distinctions between trait and ability models of emotional intelligence.

## 1. Introduction

In 1990, two distinct but potentially related concepts began to attract attention: behavioural addictions and emotional intelligence (EI), coined by Goodman [1] and Marks [2] and Salovey and Mayer [3], respectively. As a result, research on these concepts increased, particularly in the last decade. Yet still, not every aspect of the scientific inquiry of these subjects has been systematised. This problem is a consequence of shortcomings in both basic (e.g., declarative definition of a term) and further steps (e.g., operationalisation and assessment of a concept) of the scholar investigations. Therefore, in addition to reporting on the existing literature on EI and its relationship with behavioural addictions, the present review aims to identify the gaps in research practice on these topics in order to guide future work.

Behavioural addictions, despite lacking a formally acknowledged definition, are conceptualised based on observations on excessive involvement in an activity with tolerance, withdrawal, loss of control, craving, cognitive salience, or mood regulation [4]. Although gambling disorder has been reclassified as a behavioural addiction, within the substance-related and addictive disorders in the fifth edition of DSM [5], during the last decades other categories of behavioural addictions have emerged in relation to developing technologies (e.g., internet, smartphone, and/or social media) and other types of activities, such as eating, exercising, shopping, working, and sex, performed in an excessive sense. Robins and Clark [6] argued that to define if a behaviour is an addiction or not, goals behind performing a behaviour and its consequences should be examined. For instance, while some motives for excessively performing a behaviour may be due to needs and desires for sociability, to love and to be loved, procure a meaningful position within the human herd (e.g., competence, self-assertion), play, and imitate/learn, possible consequences of behavioural addictions can be emotional distress, substantial debts, marital conflict, social isolation, and impairment in job performance [7]. Several studies [8,9,10,11,12] have reported the association between behavioural addictions and deficits in emotion regulation, which is a set of strategies selected and implemented to guide the awareness of one’s own emotions and choice of following behavioural responses [13]. As a result, there has been an increased interest in investigating the potential role of EI, of which emotion regulation is a major component, in the study of behavioural addictions. However, there are important considerations when investigating the concept of EI.

Grounded work on EI describes it from two distinct perspectives: EI as a cognitive ability and as a personality trait. It is initially defined as the ability to solve a problem in an emotion-related context through four stages: perceiving emotions accurately in oneself and others, understanding those emotions, regulating them, and strategically utilising them in oneself and others [3,14]—formulised as The Four Branch Model of EI (i.e., the ability model). It is claimed to be a related concept to intelligence quotient (IQ); a cognitive ability that is measured through maximal performance items (e.g., like IQ tests). On the other hand, Petrides and Furnham [15] proposed trait EI, also called trait emotional self-efficacy, as a constellation of emotion-related self-perceptions and dispositions, assessed through self-report, unlike how ability EI is intended to be measured. While the ability model has four main components as mentioned above, trait EI, as a personality construct, is composed of four main factors (i.e., emotionality, self-control, well-being, and sociability) and fifteen different facets (e.g., emotion regulation, self-esteem, impulse control, adaptability). For detailed information on both models, the following references can be checked: [16,17].

In brief, it is imperative to consider that the conceptualisation and measurement of EI in the two models are different; therefore, they should be explored and interpret differently. However, this important point is often overlooked, resulting in research studies with misleading results on a topic of increasing interest. Given the clear link between emotion regulation and behavioural addictions, the present systematic review aims to uncover the international evidence from studies investigating the role of EI, as defined by two different conceptualisations (i.e., ability EI and trait EI), on behavioural addictions; identify the results of divergent EI models; highlight the important considerations in the study of EI to inform future research.

Although a couple of reviews [18,19] have already been conducted on the role of EI in addictions, the current review differs from the previous ones for a number of reasons. Firstly, both studies focused on addictions in general, whilst our work specifically targets behavioural addictions. Secondly, the coverage of date ranges for the search results changes in each study: 1990–2009 [18]; 2009–2016 [19]; 2013–2024 in our study. Thirdly, especially with the latest conducted review [19], there are also differences in the information sources (i.e., databases) used and the eligibility criteria for the study selections; our review places emphasis on both models of EI measured by a number of selected EI tools, differing from the previous work, which is conducted in different databases and with a focus on a single EI tool.

## 2. Materials and Methods

The reporting of this systematic literature review followed the 2020 version of Preferred Reporting Items for Systematic reviews and Meta-Analyses (PRISMA) guidelines [20].

### 2.1. Information Sources

Searches were performed in PsycArticles, PscyINFO, PubMed, Scopus, and Web of Science in August 2024 using the following keywords (30): emotional intelligence, behavioral addiction, behavioural addiction, internet addiction, compulsive internet use, compulsive information seeking, addictive internet behavior, addictive internet behaviour, problematic internet use, internet gaming disorder, social media addiction, problematic social media use, instagram addiction, problematic instagram use, tik tok addiction, digital device addiction, smartphone addiction, pathological gambling, internet gambling addiction, compulsive shopping, compulsive buying, impulsive buying, work addiction, workaholism, sex addiction, sexual addiction, cybersexual addiction, eating problems, food addiction, binge eating disorder. Search details for each database are available in Table S1.

### 2.2. Eligibility Criteria

The inclusion criteria for the search strategy were as follows: articles published in (a) English, (b) as journal articles, and (c) between 2013 and 2024. The inclusion criteria for the study selection were (a) a focus on the aforementioned keywords on behavioural addictions and (b) measuring EI with most widely used and validated tools of the ability and trait models, which are the MSCEIT (Mayer-Salovey-Caruso Emotional Intelligence Test) [21], SREIT (Schutte Self Report Emotional Intelligence Test) [22], TEIQue (Trait Emotional Intelligence Questionnaire) [23], TMMS (Trait Meta-Mood Scale; TMMS-24—Trait Meta-Mood Scale 24-item) [24], and WLEIS (Wong and Law Emotional Intelligence Scale) [25].

Search results that were not published in English and were non-journal documents (e.g., book chapters, dissertations, posters) were excluded. Other systematic literature reviews, meta-analyses, and studies that used an EI tool that either aimed to measure mixed models of EI or had unknown references were excluded. The reason for excluding mixed models is that it is a controversial area that is beyond the focus of this review.

### 2.3. Search Strategy and Data Extraction

**Figure 1:**
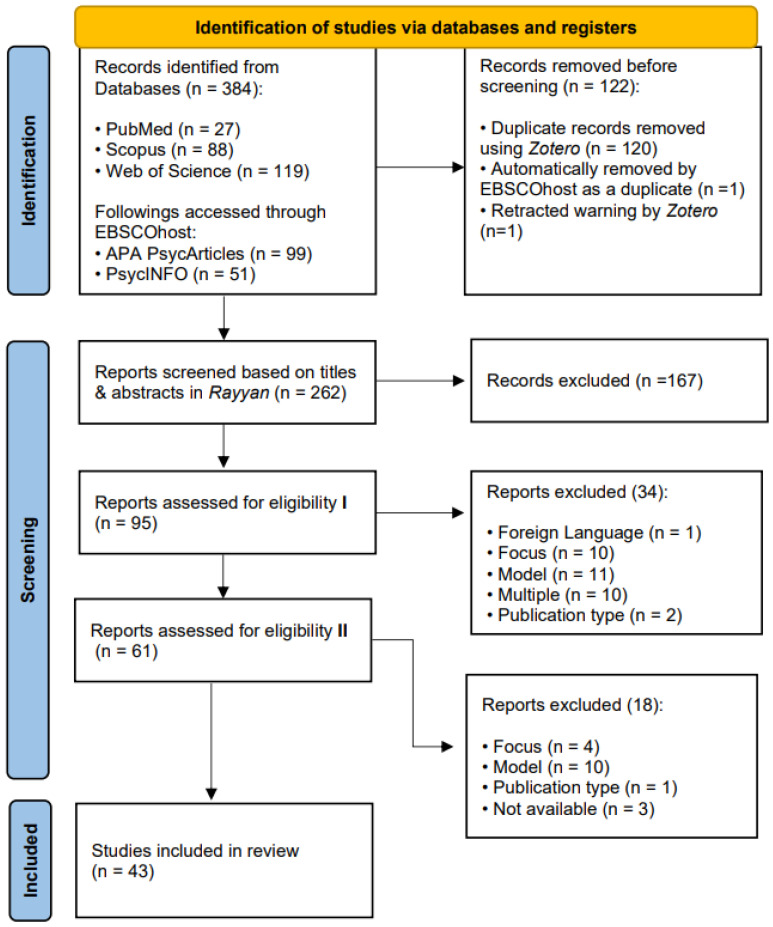
PRISMA flow diagram of the study selection process.

The search identified 384 studies. The study selection through an extensive data extraction process is displayed in Figure 1. First, several duplicate results (122) were removed using Zotero 6.0.27 [26]. Second, a three-step data screening took place with the collaboration of all authors, both using Rayyan [27] and manually on a screening sheet prepared by the second author. Finally, 43 studies were included in this systematic literature review, meeting all the eligibility criteria that we had established.

### 2.4. Risk of Bias Assessment of the Reviewed Studies

In this systematic review, sources of biases were assessed via an adapted Effective Public Health Practice Project (EPHPP) tool [28]. Table 1 briefly describes the components of this assessment. Details regarding these components for each individual study can be seen in the Table S3.

### 2.5. Organisation of the Results

In Section 3, the findings are reported under four main subtitles as follows: (Section 3.1) technology-related behavioural addictions: internet, smartphone, and social media (Section 3.2); internet gaming disorder (Section 3.3); eating disorders (Section 3.4); consumer behaviour and compulsive buying. The results of the included articles are summarised in Table S2 with the inclusion of the following information: study focus, design, sample description, EI tool, statistical methods, summary of EI-focused findings, and corresponding statistics. Table S2 is divided into two sections to separately report the results from different models of EI. However, it is important to note that only the second part of this table (i.e., trait EI results) could be consistently reported due to the shortcomings mentioned above. To recall, the main difference between the different models of EI lies in their conceptualisation and measurement. Ability EI is claimed to be measured via performance items (like IQ tests), not through self-report (like trait EI). However, several widely used EI tools, such as the SREIT and WLEIS, are constructed based on the ability model, but are self-report measures. This challenges the idea of EI described as a cognitive ability. Nevertheless, Table S2 is reported with respect to the statements made by the test developers (e.g., results from the SREIT and WLEIS are reported under the ability EI section). It is important to note that self-reported EI cannot represent EI described as a cognitive ability. For this reason, we preferred reporting “ability EI” results as “EI measured by [the tool name]” in the section below.

## 3. Results

Forty-three studies are reviewed to identify the role of emotional intelligence (EI), defined both as an ability and as a personality trait, on behavioural addictions. While most of the reviewed studies were focused on technology-related behavioural addictions, a few of them were focused on gaming (5), eating disorders (5), and consumer behaviour (4). Studies focusing on technology-related behavioural addictions, such as internet addiction and problematic social media use, dominated the area. Our application-specific search terms (e.g., Instagram addiction, problematic Instagram use, TikTok addiction) and other kinds of behavioural addiction categories (e.g., work addiction, sex addiction, cybersexual addiction) did not appear in our search results. That is, the role of EI in these concepts has not yet been the subject of research.

### 3.1. Technology-Related Behavioural Addictions: Internet, Smartphone, and Social Media

Most of the reviewed literature focused on technology-related behavioural addictions, whose conceptualisation is still debated in the literature. Therefore, many of the studies summarised here reported on emerging themes in several interchangeably used phrases, such as internet addiction (IA), problematic internet usage, addictive internet behaviours, problematic social media use, social network addiction, smartphone addiction, and addictive smartphone behaviour.

A before–after study [29] investigated IA and its influence on the personality and anxiety state of adolescents by testing the efficiency of a training programme, which was designed to improve EI. Interestingly, EI measured by a little-known test (i.e., Lusin’s) showed significant positive correlations between EI and IA. Although Lusin’s test is not in our selection criteria, we kept this study as they also used the TMMS. EI scores also measured by the TMMS scale were increased after the training. However, there was not detailed information on the training plan, Lusin’s EI measure, or statistics on the results; hence, it is difficult to draw definite conclusions from this study. The rest of the studies investigating the correlations between EI and IA were cross-sectional and reported that EI measured by the SREIT [30,31], BEIS-10 [32] (a modified version of the SREIT) [33], WLEIS [34,35], and TMMS-24 [36] was negatively correlated with IA. It was also concluded that EI had a direct effect on reducing IA [32], a negative influence on IA [37], and the component of emotion regulation partially mediated the effect of IA on negative mood but EI overall did not moderate the effect of IA on negative mood [34].

Studies on problematic/pathological internet usage (PIU) reported that EI measured by the WLEIS was negatively correlated with PIU [38,39,40], moderated the relationship between PIU and suicidal ideation [38] as well as peer attachment, perceived rejection, and PIU [40], and negatively correlated with cyberbullying [39] and cyberbullying perpetration, which is also predicted by EI and PIU relationship [41]. One study specifically focusing on EI, defined as a personality trait and measured via the TEIQue-SF [42], reported that the self-control facet of trait EI (composed of three factors: emotion regulation, impulsiveness, and stress management) was the strongest significant and negative predictor of PIU. Trait EI was also reported to be negatively correlated with addictive internet behaviours [43] and had a negative significant effect on PIU [44].

Problematic social media use (PSMU) was reported to be inversely related to trait EI [45,46,47] via motives of expressing or presenting a popular self and passing time [45]. Furthermore, trait EI was also negatively correlated with fear of missing out (FOMO), which mediates the association between PSMU, the dark triad, and trait EI [46]. EI measured via the WLEIS was negatively correlated with PSMU, and their association was mediated by the perceived stress and depressive symptoms [48]. Jarrar et al. [49], who conducted a study on late adolescents reported that EI measured via the SREIT was negatively correlated with social media addiction; entertainment and relationship maintenance as the reasons of social media use mediated the relationship between EI and social media addiction. Another recent study also conducted on adolescents found that low levels of trait EI predict the likelihood experience of social network addiction [50]. Only one study [51] focused on platform-specific problematic use: Facebook intrusion. The results showed that EI, measured by a Polish adaptation of the SREIT, was weakly correlated with Facebook intrusion. While the emotion regulation component was positively correlated with Facebook intrusion, appraisal and the expression of emotion had an indirect effect on it via anxiety and insomnia [51].

A number of studies were conducted on smartphone usage. While one study showed that EI measured by the SREIT was weakly but positively correlated with addictive smartphone behaviour [52], other studies provided evidence on the negative correlation between smartphone addiction and EI measured by the WLEIS [53,54], which also had a negative direct effect on smartphone addiction, and together with FOMO, mediated the relationship between perceived social support and smartphone addiction [55], and partially mediated the relationship between COVID-19 victimisation experience (i.e., negative thoughts and psychological trauma on corona pandemic) and smartphone addiction [54]. Ergün and Güzel [56], reported that individuals who over-exercise had a lower level of utilisation of emotions (i.e., flexible planning, creative thinking, redirected attention, and motivation [22]) and a high level of smartphone overuse. Finally, three recent studies, in which ability EI was measured via the WLEIS, reported that EI was negatively correlated with use, abuse, and addiction to the smartphone and smartphone applications [38,57,58]. In the low levels of EI, a significant conditional indirect effect of psychological distress on PSU via rumination was observed [58].

### 3.2. Internet Gaming Disorder

While gambling disorder is the only behavioural addiction taking place in the DSM-5, internet gaming disorder (IGD) is considered to be the closest possible type of behavioural addiction to follow gambling disorder [6]. In the reviewed study, only five studies focused on gaming; four conceptualised it as IGD [59,60,61,62,63], one as online gaming addiction [64]. Only two studies [61,62] were consistent with the theory and measurement of EI by focusing on trait EI defined by Petrides [15] and measured via the TEIQue-SF [23]. Generally, focus was on adolescent samples. The results showed that trait EI is negatively correlated with IGD [60,61,62,63,64]. Trait EI was also indirectly associated with problematic online gaming via mindfulness and rumination [47]. Torres-Rodriguez et al. [59] did not find correlations between self-reported IGD and EI scores, measured by the TMMS-24. These results also demonstrate how EI measured by different tools may provide different results.

### 3.3. Eating Disorders

In the reviewed literature, only four studies focused on eating disorders, including bulimic symptoms [65], binge eating [66], and eating disorders (in generic terms) [67,68]. None of these studies focused on the relationship between eating disorders and EI from a developmental perspective. That is, there was no specific focus on children, adolescents, or adults separately; instead, there were participants from a wide range of ages. As in the previous section on IGD, only two studies [66,68] were consistent with the theory and measurement of EI. For the measurement of ability, the model by Gardner et al. [65] was the only study in the reviewed literature where the MSCEIT was used, which is an ability EI tool developed by the pioneers of ability EI theory, and it is constructed based on performance items, not a self-reporting scale. The results revealed that ability EI scores were negatively correlated with global bulimic symptoms [65] and eating disorders [67]. Moreover, trait EI was found to be negatively correlated with binge eating [66] and eating disorder symptoms [68] such as indicators of anorexia nervosa and bulimia. Furthermore, trait EI and body mass index association was stronger for the clinical group, compared to controls; trait EI was also lower for individuals with class III obesity (≥40.0 kg/m^2^) [66].

### 3.4. Consumer Behaviour and Compulsive Buying

Four recent studies investigated the role of EI in impulsive buying tendency (IBT) [69], impulsive online buying [70], consumer behaviour [71], and compulsive buying [72]. Based on the measures they used to assess EI, they are all grouped as studies aimed to measure ability EI (as seen in Table S2). However, it should be noted that in these studies, there is an oversight of compliance between the theoretical basis of EI and its measurement method. The results from these cross-sectional studies show that EI measured via the WLEIS was inversely correlated to IBT [69,70], mediated the relationship between dispositional mindfulness (e.g., a tendency to be mindful in everyday life) and IBT [69], and moderated the relationship between internet usage and consumer impulsive buying behaviour [70,71]. In one randomised controlled trial [72], in which EI was aimed to be improved to help consumers by reducing their level of materialism and compulsive buying (as a result of improved EI), it was reported that their intervention was successful.

## 4. Discussion and Future Lines of Research

The present review identified and synthesised forty-three studies that aimed to investigate the relationship between behavioural addictions and EI. Overall, the reviewed literature presents findings on the inverse relationship between EI and behavioural addictions, and how EI can play a valuable mediating and/or moderating role in the effect of behavioural addictions on several outcomes (e.g., depressive symptoms, suicidal ideation, FOMO, rumination). However, the predominance of cross-sectional studies (40/43) with theoretical and methodological shortcomings makes it difficult to generalise the results, requiring further research. It would be beneficial to give thoughtful attention and take deliberate steps toward implementing more robust research designs in studies addressing the relationship between EI and behavioural addiction.

In the field of behavioural addictions, there is some debate about what constitutes addiction/dependence/problematic use, partly because of the lack of commonly accepted definitions and conceptualisation; this consequently affects the methodological assessment of these concepts. However, it is also the case that the operationalisation of new behavioural addictions is a tricky matter. Billieux et al. [4] highlighted the risk of over-pathologizing everyday life activities that could be observed as excessive and suggested that individual and motivational differences, functional impairment (i.e., significant deleterious impact on the daily life), and the stability of the dysfunctional behaviour should be considered when evaluating if a behaviour is pathological or not. Most studies in the reviewed literature used questionnaires to assess behavioural addictions, which would not be the only way to assess other types of addictions (e.g., substance-use addictions) that seems to occur in similar ways. It has been suggested that neurobehavioral and neurocognitive studies may be helpful in the classification issue of behavioural additions [6]. Yet still, despite existing questionnaires, the measurement of addiction remains challenging [73] and requires further research. Collaboration between researchers and clinicians could improve efforts to understand behavioural addictions. Relationships between developmental processes, different motivations underlying addictive behaviours, and the development of behavioural addictions may also be considered. An increasing interest in technology-related behavioural addictions is an understandable situation; however, future research could also place more emphasis on other types of behavioural addictions (e.g., relating to eating, working, shopping, etc.) in their relationship with EI constructs.

In the field of EI, although clear distinctions have been made both theoretically and psychometrically between the two diverging models, ability EI and trait EI, these distinctions are still overlooked in research. This therefore tends to produce inconclusive results, potentially complicating the interpretation of EI research findings. This is particularly true when studies examine two complex topics, such as behavioural addictions and EI, both of which face descriptive and methodological limitations. Such limitations can lead to conclusions that risk misrepresenting these concepts.

Several studies in the reviewed literature that described EI as an ability and measured with a trait EI tool created a domino effect for subsequent research deficiencies such as the flawed investigation and interpretation of the concept. Two possible factors might underlie this issue: (1) insufficient attention to the distinction between the two theoretical frameworks, or (2) a reliance on a widely used tool developed for a different framework without adequately addressing this theoretical difference. For example, as mentioned earlier, ability EI, described as a cognitive ability like IQ, is proposed to be measured through performance items, not via self-reporting. In this context, the MSCEIT appears to be the only suitable choice for assessing ability EI, as it is specifically designed as a maximum performance test.

Although the SREIT and WLEIS are widely used to assess ability EI, they rely on Likert scales rather than maximum performance tests, which is inconsistent with the operationalization of ability EI model. Furthermore, two reviews on EI measures classified them differently: as an ability model [74], and as a trait-based measure [75]. It is also interesting that although the WLEIS has a specific focus on EI in leadership and management studies, it is used by researchers for a wide variety of research foci such as behavioural addictions. Additionally, there is a simple but confusing problem with the reporting of results from the SREIT in the literature, where it is referred to by many different acronyms such as SSRI, SEIS, and EIS, which may refer to different measures. To keep it consistent, we preferred to report it as SREIT, both in the text and in the main table (i.e., Table S2) when reporting results from Schutte’s test [22].

Lastly, two widely used EI measures we included in this review were the TMMS and TEIQue. The TMMS is a self-report scale, developed by the ability model pioneers, designed to measure emotional attention, clarity, and repair (i.e., not ability EI as a whole but just an aspect of it). Again, as it is not a maximal performance test, it is tricky to classify it as an ability model measure. Finally, the TEIQue, as a validated trait EI measure, is the leading measurement tool for the trait EI theory. Although studies included in this review used only one version of it (i.e., TEIQue Short Form), the TEIQue has several forms that can be specifically used with child, adolescent, and adult samples. That is, it provides validated tools to assess EI in different developmental groups. One study [66] used another trait EI form (i.e., MEIA [76]) that is claimed to be a “purer” measure of the construct. However, this, again, could be open to discussion. It is based on the ability model; therefore, factors of it differ from the trait EI model proposed by Petrides and Furnham [15]. Selecting an EI tool that aligns with the EI model that is aimed to be explored is a crucial step toward ensuring accurate and meaningful results. By prioritising this alignment, future research can advance the field of EI with greater clarity and reliability.

Regarding risk of bias assessment results (as shown in Table S3), most of the studies have low and medium quality. According to the EPHPP tool, one weak rating among the various components listed in Table 1 results in a medium overall rating. Because most of the studies had a cross-sectional design, this meant that many of them were rated as weak or moderate, regardless of the other strengths and limitations they had.

As discussed in detail, the reviewed literature on the relationship between EI and behavioural addictions reveals several areas that would benefit from refinement. These include aligning the measurement methods with the theoretical framework of EI, exploring diverse research designs beyond cross-sectional studies, and finding ways to complement self-reporting with additional assessment tools to enhance reliability. Furthermore, greater attention to the transparent and detailed reporting of study methodologies and corresponding statistical analyses would contribute to the robustness of the findings. Addressing these aspects can provide a stronger foundation for advancing our understanding of the interplay between EI and behavioural addictions, because regardless of the risk of bias the current literature has, the role of EI, defined in both forms—as ability and trait—in psychopathological outcomes and addictions remain undoubtedly protective and valuable to investigate, as the core of EI is emotion regulation, which is a key concept in the development and presence of addictions. Through scientific research that is as transparent and reliable as possible, it is also important to deepen the construct for clinicians working with clients with behavioural addictions. A clear understanding of the relationship between EI and behavioural addictions, with supporting scientific evidence, would facilitate the design of appropriate interventions to increase EI in the clinic and in addiction prevention.

In addition to the shortcomings of the revised literature, this review itself also has a few limitations: one unmet PRISMA statement (i.e., protocol registration), the restrictions we placed on our search and selection criteria (i.e., including only journal articles published in English), which inevitably introduced language bias, and perhaps a loss of information as we excluded grey literature. However, our detailed discussion identifies areas for improvement in the reviewed literature, with the goal of guiding future research toward more robust and successful outcomes.

## 5. Conclusions

The current review presents studies from the last decade that examine the relationship between EI and behavioural addictions. The majority of the results show that EI is negatively correlated with behavioural addictions and plays both a moderating and mediating role in the associations between behavioural addictions and other negative outcomes. However, the limitations of research in both areas make it difficult to draw comprehensive conclusions. This paper also aimed to provide a detailed discussion of these limitations in order to provide guidance for future research on EI and behavioural addictions. Flawless research may not be possible, but only rigorous designs can give us real clues to explore an issue where we want to make a difference through research, training programmes, and interventions to optimise EI and prevent behavioural addictions.

## Figures and Tables

**Table 1:** Adapted EPHPP tool content (Effective Public Health Practice Project, 1998).

Component	Description
Selection Bias	The extent to which participants are representative of the target population.
Study Design	Type of study and randomisation process.
Blinding	Awareness of the outcome assessor and study participants.
Data Collection Methods	Validity and reliability of data collection tools.
Analyses	Appropriateness of the statistical methods chosen for analysis.

## Data Availability

All extracted data are available upon request.

## Supplementary Materials

The following supporting information can be downloaded at: https://www.mdpi.com/article/10.3390/jcm14041125/s1, Table S1. Search details on different databases; Table S2. Summary of the reviewed studies; Table S3. Quality Assessment of the included studies based on Adapted EPHPP (Effective Public Health Practice Project, 1998) tool.

## Abbreviations

BEIS-10	Brief Emotional Intelligence Scale
EI	Emotional Intelligence
FOMO	Fear of Missing Out
DSM-5	The Diagnostic and Statistical Manual of Mental Disorders, Fifth Edition
IDG	Internet Gaming Disorder
IA	Internet Addiction
IBT	Impulsive Buying Tendency
IGD	Internet Gaming Disorder
IQ	Intelligence Quotient
PIU	Problematic Internet Use
PRISMA	Preferred Reporting Items for Systematic reviews and Meta-Analyses
PSMU	Problematic Social Media Use
SREIT	Schutte Self Report Emotional Intelligence Test
TEIQue	Trait Emotional Intelligence Questionnaire
TEIQue-SF	Trait Emotional Intelligence Questionnaire Short Form
TMMS	Trait Meta-Mood Scale
TMMS-24	Trait Meta-Mood Scale-24 item
WLEIS	Wong and Law Emotional Intelligence Scale
