# Supplementary material for: Emotional Intelligence and Behavioural Addictions: A Systematic Review
Source: J Clin Med. 2025 Feb 10;14(4):1125. doi: 10.3390/jcm14041125 (PMC11856372; doi:10.3390/jcm14041125)
Supplement: Supplementary file 1 [file jcm-14-01125-s001.zip › jcm-3378567-supplementary.pdf]

Supplementary Table S1.

Search details on different databases.

Database	Search code
PsycArticles & PscyINFO[†]	TX "emotional intelligence" AND ("behavioral addiction" OR " behavioural addiction" OR “internet addiction” OR “compulsive internet use” OR “compulsive information seeking” OR “addictive internet behavior” OR “addictive internet behaviour” OR “problematic internet use” OR “internet gaming disorder” OR “social media addiction” OR “problematic social media use” OR “instagram addiction” OR “problematic instagram use” OR “tik tok addiction” OR “digital device addiction” OR “smartphone addiction” OR “pathological gambling” OR “internet gambling addiction” OR “compulsive shopping” OR “compulsive buying” OR “impulsive buying” OR “work addiction” OR “workaholism” OR “sex addiction” OR “sexual addiction” OR “cybersexual addiction” OR “eating problems” OR “food addiction” OR “binge eating disorder”)
PubMed[‡]	(("emotional intelligence"[Title/Abstract]) AND (("behavioral addiction"[Title/Abstract]) OR ("behavioural addiction"[Title/Abstract]) OR ("internet addiction"[Title/Abstract]) OR ("compulsive internet use"[Title/Abstract]) OR ("compulsive information seeking"[Title/Abstract]) OR ("addictive internet behavior"[Title/Abstract]) OR ("addictive internet behaviour"[Title/Abstract]) OR ("problematic internet use"[Title/Abstract]) OR ("internet gaming disorder"[Title/Abstract]) OR ("social media addiction"[Title/Abstract]) OR ("problematic social media use"[Title/Abstract]) OR ("instagram addiction"[Title/Abstract]) OR ("problematic instagram use"[Title/Abstract]) OR ("tik tok addiction"[Title/Abstract]) OR ("digital device addiction” [Title/Abstract]) OR ("smartphone addiction"[Title/Abstract]) OR ("pathological gambling"[Title/Abstract]) OR ("internet gambling addiction"[Title/Abstract]) OR ("compulsive shopping"[Title/Abstract]) OR ("compulsive buying"[Title/Abstract]) OR ("impulsive buying"[Title/Abstract]) OR ("work addiction"[Title/Abstract]) OR ("workaholism"[Title/Abstract]) OR ("sex addiction"[Title/Abstract]) OR ("sexual addiction*"[Title/Abstract]) OR ("cybersexual addiction "[Title/Abstract]) OR ("eating problems"[Title/Abstract]) OR ("food addiction"[Title/Abstract]) OR ("binge eating disorder"[Title/Abstract])))
Scopus[§]	((TITLE-ABS-KEY (" emotional intelligence")) AND (TITLE-ABS-KEY (" behavioral addiction") OR TITLE-ABS-KEY ("behavioural addiction") OR TITLE-ABS-KEY ("internet addiction") OR TITLE-ABS-KEY ("compulsive internet use") OR TITLE-ABS-KEY (" compulsive information seeking") OR TITLE-ABS-KEY (" addictive internet behavior ") OR TITLE-ABS-KEY (" addictive internet behaviour ") OR TITLE-ABS-KEY (" problematic internet use ") OR TITLE-ABS-KEY ("internet gaming disorder") OR TITLE-

	ABS-KEY ("social media addiction") OR TITLE-ABS-KEY (" problematic social media use ") OR TITLE-ABS-KEY (" instagram addiction ") OR TITLE-ABS-KEY (" problematic instagram use ") OR TITLE-ABS-KEY (" tik tok addiction ") OR TITLE-ABS-KEY (" digital device addiction ") OR TITLE-ABS-KEY (" smartphone addiction ") OR TITLE-ABS-KEY (" pathological gambling ") OR TITLE-ABS-KEY (" internet gambling addiction ") OR TITLE-ABS-KEY (" compulsive shopping ") OR TITLE-ABS-KEY (" compulsive buying ") OR TITLE-ABS-KEY (" impulsive buying ") OR TITLE-ABS-KEY (" work addiction ") OR TITLE-ABS-KEY (" workaholism ") OR TITLE-ABS-KEY (" sex addiction ") OR TITLE-ABS-KEY (" sexual addiction ") OR TITLE-ABS-KEY (" cybersexual addiction ") OR TITLE-ABS-KEY (" eating problems ") OR TITLE-ABS-KEY (" food addiction") OR TITLE-ABS-KEY (" binge eating disorder "))) AND PUBYEAR > 2012 AND PUBYEAR < 2025
Web of Science[††]	TS = (("emotional intelligence") AND ("behavioral addiction" OR " behavioural addiction" OR “internet addiction” OR “compulsive internet use” OR “compulsive information seeking” OR “addictive internet behavior” OR “addictive internet behaviour” OR “problematic internet use” OR “internet gaming disorder” OR “social media addiction” OR “problematic social media use” OR “instagram addiction” OR “problematic instagram use” OR “tik tok addiction” OR “digital device addiction” OR “smartphone addiction” OR “pathological gambling” OR “internet gambling addiction” OR “compulsive shopping” OR “compulsive buying” OR “impulsive buying” OR “work addiction” OR “workaholism” OR “sex addiction” OR “sexual addiction” OR “cybersexual addiction” OR “eating problems” OR “food addiction” OR “binge eating disorder”))

Note: In all databases, if not indicated in the search code, date range and language is manually selected as 2010-2024 and as English, respectively.

[†] PsycArticles and PscyINFO were searched through EBSCOhost with the same search code. Non article content, book reviews and dissertations are excluded manually; following expanders are used: apply related words, also search within the full text of the articles, apply equivalent subjects.
[‡] Phrases “compulsive information seeking, addictive internet behaviour”, “tik tok addiction”, and “digital device addiction” were not found.
[§] Source type was chosen “Journal” (i.e., Conference proceedings (2) and a book was excluded by choosing this).
[††] TS in Web of Science refers to Topic; runs the search in title, abstract and author keywords.

Supplementary Table S2.

Summary of the reviewed studies.

	Study	Focus	Design	Sample Description (female%, Age range, M_{age})	EI Tool	Statistical Analysis	Summary of Findings†	Statistics
	Ability EI							
[57]	Aranda et al. (2022)	PIU, PSU	Cross-sectional	522 participants (74.1%, 18-35, 21.79)	WLEIS	Stepwise multiple linear regression analysis, one-way and univariate ANOVAs	Ability EI (a) dimensions were negatively correlated with use, abuse, and addiction to the smartphone and its applications; (b) dimension –evaluation of one's own emotions, slightly explained the variability of the participants' problematic smartphone usage.	(a) r = -.23** (evaluation of own emotions)<br>r = -.04 (evaluation of others' emotions)<br>r = -.13** (use of emotions)<br>r = -.24** (regulation of emotions)<br>(b) β = -.097*<br>t = -2.43<br>(R^2_{adj} = .25<br>SE = .52<br>ΔR^2 = .01<br>F (4,546) = 46.85<br>p < 0.001)
[38]	Arrivillaga et al. (2020)	PIU, PSU	Cross-sectional	2196 participants (54%, 12-19, 14.60)	WLEIS	Correlation analysis, simple moderation analysis model	Ability EI (a) was negatively correlated with PIU and PSU; (b) moderated the relations between PIU/PSU and suicidal ideation, (c) especially in low AEI participants.	(a) r = -.18** (PIU)<br>r = -.14** (PSU)<br>(b) b = -.09** SE = .03 ΔR^2 = .004** (PIU)<br>b = -.04** SE = .02 ΔR^2 = .003* (PSU)<br>(c) b = .16, $t_{(1944)}$ = 6.06*** (PSU)
[48]	Arrivillaga et al. (2022)	PSMU	Cross-sectional	2068 participants (53.8%, 12-19, 14.61)	WLEIS	Pearson bivariate correlations, Harman's single	Ability EI (a) was negatively correlated with perceived stress, depressive symptoms, and PSMU; (b) and PSMU	(a) r = -.40** (perceived stress)<br>r = -.30** (depressive symptoms)<br>r = - .14** (PSMU)

						factor test, mediation analyses	association was mediated by the perceived stress and depressive symptoms; (c) to PSMU path was sequentially mediated by perceived stress and depressive symptoms.	(b) Boot indirect effect = -.78 BootSE = .12, 95% CI [-1.04, -.55] (perceived stress); Boot indirect effect = -.11, BootSE = .04, 95% CI [-.20, -.04] (depressive symptoms) (c) Boot indirect effect = -.23, BootSE =.06, 95% CI [-.35, -.12]
[58]	Arrivillaga et al. (2022)	PSU	Cross-sectional	1882 participants (54% female, 12-19, 14.71)	WLEIS	Moderated mediation analysis	Ability EI (a) was negatively correlated with PSU; (b) the mediation of psychological distress on PSU by rumination was moderated by EI, controlling for age and gender; (c) the conditional indirect effect of psychological distress on PSU via rumination was significant at low, but not at high levels of EI.	(a) r = -.13** (b) b = − .05, SE (HC3) = .02, 95%CI [− .106, − .004] (c) Low EI (M-SD) = -1.0047, Boot Indirect Effect = .1092, Boot SE = .0261, Boot 95%CI [.0589, .1618]; High EI(M-SD) = 1.0047, Boot Indirect Effect = .0438, Boot SE = .0223, Boot CI [-.0005, .0890]
[51]	Błachnio et al. (2023)	Facebook intrusion	Cross-sectional	1396 participants (71%, 16-NA, 21.25)	INTE‡	Pearson's correlation, SEM, mediation analysis	Ability EI (a) was weakly correlated with Facebook intrusion; (b) scores slightly differ among males and females; (c) -regulation of emotion- was positively and significantly related to Facebook intrusion; (d) -appraisal and expression of emotion - had an indirect effect on Facebook intrusion via anxiety and insomnia.	(a) r = .01 (b) *d* = .27** (c) χ^2 (24) = .43, CFI = 1, RMSEA = .001 (model 1): β = 0.13***, 95% CI [.05, .20] (d) β = .011, 95% CI [.001, .24], p= .023

[64]	Che et al. (2017)	Online gaming addiction	Cross-sectional	931 participants (0%, 13-19, 16.18)	Chinese Emotional Intelligence Scale§	Correlation analysis, mediation analysis	Ability EI dimensions (self-management, social skills, empathy) and online gaming addiction indicators (core symptoms, negative outcomes) were negatively correlated.	$-.232^{**} < r < -.092^{**}$
[54]	Chen & Zhang (2023)	Smartphone addiction	Cross-sectional	1154 participants (54.5%, 17-20, 18.9)	WLEIS	SEM	Ability EI (a) and smartphone addiction was negatively correlated; (b) significantly predicted smartphone addiction; (c) partially mediated the association between COVID-19 victimization experience and smartphone addiction.	(a) $r = -.231^{***}$<br>(b) $\beta = -.0186^{***}$<br>(c) $\beta = -.065$, 95% CI [.034, .101]
[60]	Dang et al. (2019)	IGD	Longitudinal	469 participants (time 1) (58%, 18-27, 19.29)<br><br>282 participants (time 2) (60%, 19-28, 20.47)	WLEIS	Attrition analyses, correlation analyses, path analysis	**Trait EI**†† (a) was negatively correlated with IGD at both times; (b) had a significant indirect effect on IGD tendency through depression and, coping flexibility and depression.	(a) $r = -.23^{*}$ (time 1)<br>$r = -.14^{*}$ (time 2)<br>(b) $\beta = -.08$, 95% CI [-.14, -.03], $p < .001$ (depression)<br>$\beta = -.05$, 95% CI [-.08, -.02], $p < .001$ (coping flexibility and depression)
[56]	Ergün & Güzel (2019)	PSU	Cross-sectional	778 participants (56.9%, 18-64, NA‡‡)	SREIT	Chi-square test, ANOVA	Ability EI factor -utilization of emotions- was lower for gym-goers who have exercise dependence.	M = 18.34 SD = 4.05 (Asymptomatic)<br>M = 17.89 SD = 3.2 (Symptomatic)<br>M = 17.05 SD = 3.8 (Dependent)<br><br>$F(3,775) = 3.86^{*}$

[36]	Fernández-Martínez et al. (2023)	IA	Cross-sectional	530 participants (85.2%, 17-20, 18.9)	TMMS-24	Cronbach's alpha, non-parametric tests, simple logistic regression	Ability EI (a) factors emotional clarity and emotional repair has weak negative correlations with IA; (b) increase in attention items is associated with increase in IA scores, however, increase in emotional clarity items is associated with decrease in the IA scores.	(a) r = -.169** (emotional clarity) r = -.095* (emotional repair) (b) R^2 = .014 O.R = .089 95%IC [1.041, 1.114] p = .001 (attention); R^2 = .034 O.R = -.080 95%IC [.879, .969] p = .000 (emotional clarity)
[67]	Foye et al. (2019)	Eating Disorders	Cross-sectional	355 participants (84%, 18-50+, NA)	SREIT	Pearson correlation, a series of Mann–Whitney U tests	Ability EI scores were negatively correlated with *The Eating Attitudes Test* scores of the participant group who had a history of an eating disorder.	r = -.371*** (overall EI)
[65]	Gardner et al. (2014) §§	Bulimic Symptoms	Cross-sectional	235 participants (100%, 18-79, 36.2)	MSCEIT	Correlational analysis	Ability EI (a) factor Managing Emotions (*in medium level EI scorers*) and (b) factor Social Management (*in low level EI scorers*) was negatively correlated with global bulimic symptoms.	(a) r = -.14* (b) r = -.19**
[35]	Hoang et al. (2023)	IA	Cross-sectional	787 participants (58.8%, NA, NA)	WLEIS	EFA, CFA, SEM	Ability is negatively associated with Internet addiction.	b = -.386 β = -.428, SE = .087 CR = -4.450, p < .001
[32]	Hsieh et al. (2019)	IA	Cross-sectional	6233 participants (49.7%, NA,	BEIS-10§§	Hierarchical linear regression	Ability EI (a) was negatively correlated with IA; (b) had direct effect on reducing IA; (c) was not found to be a	(a) r = -.18** (b) b = -.17, SE (B) = .02 β = -.13*** (Model 1); B = -.17, SE (B) = .04

				NA – fourth graders)			moderator of the relationship between peer victimization and IA.	β = -.13*** (Model 2)<br>(c) b = .00, SE (B) = .00, β = -.01
[49]	Jarrar et al. (2022)	Social Media Addiction	Cross-sectional	400 participants (52%, 18-25,)	SREIT	Path analysis, GLM	Ability EI (a) was negatively correlated with social media addiction; (b) has a negative predictive effect on it; (c) and social media addiction was mediated by entertainment and relationship maintenance as reasons of social media use.	(a) r = -.337**<br>(b) β = -.337***<br>(c) β = -.307*** (entertainment)<br>β = -.099*** (relationship maintenance)
[71]	Jie et al. (2022)	Internet Usage on Consumer Behavior	Cross-sectional	285 participants (50.9%, 20-50, NA)	WLEIS	PLS-SEM	Ability EI moderate the relationship between Internet usage and consumer impulsive buying behavior.	R^2 = .572, R^2adj = .565<br>β = .853***<br>M = .856 SD= .045<br>T = 19.105, F^2 = .423
[72]	Lekaviciene et al. (2022)	Consumer Materialism and Compulsive buying	Experimental	18 participants (cases) (NA%, 18-32, NA)<br><br>18 participants (controls) (NA%, 18-32, NA)	SREIT	Independent samples t-test	Ability EI based training programs can help consumers (a) improve their EI while (b) lowering their levels of materialism and compulsive buying.	(a) M = 3.66 SD = .26 (cases)<br>M = 3.22 SD = .35 (controls)<br>t (34) = 4.203***<br>*d* = .31<br>(b) M = 2.09 SD = .50 (cases)<br>M = 2.63 SD = .67 (controls)<br>t (34) = -2.722***<br>*d* = .59
[29]	Oskenbay et al. (2015)	IA	Before-after study	60 participants (NA%, NA, NA)	TMMS	Correlational analysis	Ability EI (a) was significantly correlated with IA; (b) scores were increased after training intervention in comparison with the baseline.	(a) r = .489**<br>(b) NA
[69]	Park & Dhandra (2017)	IBT	Cross-sectional	319 participants	WLEIS	Correlational analysis, multiple mediation analysis	Ability EI (a) was inversely correlated with IBT; (b) mediated the relationship	(a) r = -.524***<br>(b) β = - .012*

				(51.7%, NA, 28.2)			between dispositional mindfulness and IBT.	95%CI [- .19, - .03] (appraisal of emotions in oneself)<br>β = - .022*<br>95%CI [- .34, - .11] (use of emotions)<br>β = - .06*<br>95% CI [- .12, - .01] (emotion regulation)
[30]	Saraiva et al. (2018)	IA	Cross-sectional	1413 participants (57.7%, 17-65, 38.8)	SREIT	Correlational analysis, linear regression	Ability EI (a) and IA were negatively correlated; (b) dimensions of *perception of emotions* and *dealing with others' emotions* were negatively associated with IA.	(a) r = -.184***<br>R^2 = .050, R^2_{adj} = .048, B = -.396, EPB = .099, β = - .204***, t = -3.994<br>(emotion perception) (Step 2)<br>R^2 = -.329, R^2_{adj} = .100, B = -.204, EPB = -3.303, β = -.001***, t = -.329<br>(emotion management in others) (Step 2)
[59]	Torres-Rodriguez et al. (2018)	IGD	Cross-sectional	31 participants (0%, 12-18, 14.97)	TMMS-24	Correlational analysis	Ability EI was not significantly correlated with IGD scale scores.	NA
[55]	Ulwiyyah & Zhang (2022)	IA	Cross-sectional	403 participants (72.2%,17-41, 20)	WLEIS	Path analysis	Ability EI (a) had a negative direct effect on smartphone addiction; (b) and FOMO mediated the relationship between perceived social support and smartphone addiction.	(a) β = - .026***<br>(b) a*b = .02<br>Bootstrap 95% CI [-.89, -.05]
[52]	van Deursen et al. (2015)	Addictive smartphone behavior	Cross-sectional	386 participants (67%, 15-88, 35.2)	SREIT	SEM	Ability EI was weakly correlated with habitual or addictive smartphone	r = .14* (habitual)<br>r = .33* (addictive)

							behavior, and was not found to have an influence on them.	
[70]	Vihari et al. (2022)	Impulsive online buying, PIU	Cross-sectional	598 participants (33.61%, <22 - >40, NA)	WLEIS	Multiple regression, moderated mediation analysis	Ability EI (a) was negatively correlated with impulsive buying behavior and PIU; (B) was negatively moderates the relationship between PIU and online impulse buying behavior.	(a) r = -.17*** (impulsive buying behavior) r = -.34* (PIU) (b) b = -.03, 95% CI [-.04, -.01]
[37]	Vu et al. (2022)	IA	Cross-sectional	744 participants (62.2%, NA, NA)	WLEIS	Factor analysis, regression analysis	Ability EI components (3) had a negative influence on IA, except for use of emotions component.	β = -.169*** t = -4.397 (Self-emotion appraisal) β = .050 t = 1.328 (Others' emotion appraisal) β = -.117** t = -2.971 (Use of emotion) β = -.155*** t = -3.990 (Regulation of emotion)
[31]	Yekefallah et al. (2019)	IA	Cross-sectional	325 participants (74%, 18-49, 23.13)	SREIT	Pearson correlations	Ability EI was negatively correlated with IA.	r = -.163**
[34]	Yu & Zhou (2021)	IA	Cross-sectional	404 participants (47.8%, NA, 12.4)	WLEIS	SEM	Ability EI (a) components use of emotions and emotion regulation were significantly correlated with IA subscales; (b) component emotion regulation partially mediated the effect of IA on negative mood; (c) did not moderate the effect of IA on negative mood.	(a) r = -.17** (use of emotions) r = -.20** (emotion regulation) (b) β = .07, 95% CI [.03, .11] (c) β = −.09, *p* = .18
[39]	Yudes et al. (2020)	Cyberbullying, PIU	Cross-sectional	2039 participants (53.9%, 12-18, 14.6)	WLEIS	Bivariate correlation analyses, logistic regression	Ability EI dimensions were correlated negatively with (a) Cyberbullying and (b) PIU.	(a) -.14** < r < -.07** (b) -.20** < r < .005

[41]	Yudes et al. (2021)	Cyberbullying, PIU	Cross-sectional	2039 participants (53.9%, 12-18, 14.6)	WLEIS	Bivariate correlation analyses, logistic regression, moderation analyses	Ability EI was (a) correlated negatively with cyberbullying perpetration and was (b) a predictor of it; (c) and PIU relationship also predict cyberbullying perpetration.	(a) r = -.11*** (girls)<br>r = -.18*** (boys)<br>(b) β = -.279 SE = .073<br>Wald's X^2 = 14.68***<br>OR = .757<br>(c) R^2 = .116**<br>β = -.022*<br>SE = .010<br>ΔR^2 = .0019*<br>95% CI [-.43, -.001]
[63]	Zahra et al. (2020)	IGD	Cross-sectional	315 participants (48.8%, 18-25, 23.97)	WLEIS	Bivariate correlation analyses, one-way ANOVA	Ability EI and internet gaming was negatively correlated.	r = -.23***
[40]	Zhang & Wang (2021)	PIU	Cross-sectional	406 participants (50.2%, 8-16, 12.76)	WLEIS	Correlational analysis, SEM	Ability EI was (a) positively correlated with peer attachment, (b) negatively correlated with perceived personal rejection and PIU; had a moderating effect on the relationship between peer attachment (c) and perceived rejection, (d) and PIU.	(a) r = .402**<br>(b) r = -.261** (perceived personal rejection)<br>r = -.171** (PIU)<br>(c) β = -.116*<br>β = -.101*
[53]	Zhang (2023)	SPA	Cross-sectional	1.154 participants (54.5%, 17-20, 18.9)	WLEIS	Correlational analysis, CFA, SEM, bias-corrected non-para metric percentile bootstrap	Ability EI was negatively correlated with (a) SPA and (b) future anxiety, and (c) significantly predicted both of them.	(a) r = -.26***<br>(b) r = -.28***<br>(c) β = - .021*** (SPA)<br>β = - .035*** (future anxiety)
Trait EI								
[42]	Alshakhsi et al. (2022)	PIU	Cross-sectional	268 participants (61.6%, 15-64, NA)	TEIQue-SF	Pearson's correlation, One-way ANOVA, Multiple linear regression analysis	Trait EI (a) global score was moderate for the whole sampling; (b) facet Self-Control was a significant and negative	(a) M = 4.53, SD = .82<br>(b) β = -.23*** (obsession),<br>β = -.18* (neglect),<br>β = -.23*** (control disorder)<br>(c) β = -.28***

							predictor of PIU, also (c) the strongest predictor.	
[44]	Alshakhsi et al. (2023)	PIU	Cross-sectional	268 participants (61.6%, 15-64)	TEIQue-SF	Pearson's correlation, Multiple linear regression analysis	Trait EI (a) had a negative and significant effect on overall PIU, and (b) on components of PIU.	(a) β = -.35***<br>(b) β = −.29*** (obsession)<br>β = −.26***(neglect)<br>β = −.27***(control disorder)
[66]	Andrei et al. (2018)	Binge Eating	Cross-sectional	134 participants (cases) (69.4%, 23-64, 49.70)<br><br>124 participants (controls) (74.2%, NA, 40)	TEIQue–SF	Pearson's Chi-Squared, t-test, Pearson's correlations, MANOVA,	Trait EI (a) and body mass index (BMI) association was stronger for the clinical group; (b) and binge eating are negatively correlated; (c) was significantly lower for individuals with class III obesity.	(a) r = - .22*** (cases),<br>r = .07 (controls)<br>(b) r = - .29*** (cases),<br>r = -.37*** (controls)<br>(c) M = 4.6, SD = .94 (Obese class III)<br>M = 5.2, SD = .78 (Normal weight)<br>p = .004
[46]	Barberis et al. (2023)	PSMU	Cross-sectional	788 participants (75%, 18-35, 24.22)	TEIQue–SF	SEM	Trait EI (a) was negatively correlated with PSMU, dark triad (DT), and fear of missing out (FOMO), (b) which indirectly mediated the association among these variables.	(a) r = -.24** (PSMU)<br>r = -.10** (DT)<br>r = -.29** (FOMO)<br>(b) β = -.22, SE = .04, 95% CI [-.15, -.22] (Trait EI → PSMU via FOMO)
[68]	Biolcati et al. (2021)	Eating Disorders	Cross-sectional	394 participants (79.1%, 18-65, 32.34)	TEIQue–SF	ANOVA, Pearson's correlational analyses, multiple linear regression	Trait EI was negatively correlated with eating disorder symptoms and alexithymia.	r = -.30 (eating disorder)<br>r = -.72** (alexithymia)
[65]	Gardner et al. (2014) §§	Bulimic Symptoms	Cross-sectional	235 participants	MEIA	Correlational analysis	Trait EI factor Self-orientation (*in medium level EI scorers*) and factor Recognition of Emotion	r = -.32*** (Self-orientation)<br>r = -.30*** (Recognition of Emotion in self)

				(100%, 18-79, 36.2)			in self (*in low level EI scorers*) was negatively correlated with global bulimic symptoms.	
[61]	Kırcaburun et al. (2020)	IGD	Cross-sectional	478 participants (NA%, 14-38, 20.88)	TEIQue-SF	Pearson correlation tests, CFA, path analysis	Trait EI is (a) inversely related with IGD and (b) affects gaming for different motives among adolescent and adult gamers.	(a) r = -.41***<br>Cronbach's α = .77<br>(b) β = -.41***, SE = .04 (total effect in total sample)<br>β = -.20**, SE = .06 (direct effect in total sample)<br>β = -.21**, SE = .04 (total indirect effect in total sample)
[47]	Kırcaburun et al. (2019)	PSMU, POG	Cross-sectional	470 participants (59,5%, 14-18, 16.3)	TEIQue-SF	Multiple mediation model	Trait EI (a) was negatively correlated with PSMU and POG; (b) was indirectly associated with PSMU via mindfulness, rumination, and depression, and (c) with POG via mindfulness and rumination.	(a) r = -.33*** (PSMU)<br>r = -.13** (POG)<br>(b) β = -.18***, SE = .04 (via mindfulness)<br>β = -.06***, SE = .02 (via rumination)<br>β = -.04**, SE = .02 (via depression)<br>(c) β = -.18***, SE = .01 (via mindfulness)<br>β = .04**, SE = .01 (via rumination)
[62]	Ko et al. (2021)	IGD	Cross-sectional	69 participants (cases) (21.7%, 20-38, 25.32)<br><br>138 participants (controls) (21.7%, 20-38, 25.73)	TEIQue-SF	Chi-square analysis, logistic regression analysis	Trait EI (a) was lower for participants with IGD, especially in Well-being and Self-control facet, (b) which are also the most strongly associated facets with IGD.	(d) M = 4.04 SD = 1.07 (cases)<br>M = 4.97 SD = .94 (controls)<br>t = -6.34*** (Well-being)<br>M = 3.98 SD = .78 (cases)<br>M = 4.81 SD = .77 (controls)<br>t = -7.29*** (Self-control)<br>(e) Wald's X^2 = 17.55***, Exp(β) = .30, 95% CI [.17, .52] (Self-control)<br>Wald's X^2 = 8.17**, Exp(β) = .55, 95% CI [.36, .83] (Well-being)

[50]	Pino & Mastromarino (2023)	Social network abuse / addiction	Cross-sectional	226 participants (64.6%, 16-18, 17)	TEIQue-SF	Paired-samples t-test, multivariate logistic regression	Trait EI, in low levels, predicted the possibility to experience in a social network addiction.	β = -.014, SE = .007, p = 0.054, OR = 0.99, Pseudo R^2 = .15
[43]	Sechi et al. (2021)	Addictive internet behaviors	Cross-sectional	362 participants (55.8%, 16-68, 29.10)	TEIQue-SF	SEM, path analysis	Trait EI (a) was negatively correlated with addictive internet behaviors; (b) has a negative influence on addictive internet behaviors.	(a) r = -.47***<br>(b) Boot Direct Effect = -.46*** Boot CI [-.53, -.39]
[45]	Süral et al. (2019)	PSMU	Cross-sectional	444 participants (75%, 18-43-20.45)	TEIQue-SF	Pearson correlation tests, path analysis	Trait EI (a) was inversely related with PSMU; (b) was directly and indirectly associated with PSMU via motives of expressing or presenting a popular self and passing time.	(a) r = -.45***<br>(b) β = -.39***, SE = .04, 95% CI [-.46, -.31] (direct effect); β = -.07**, SE = .02, 95% CI [-.11, -.03] (indirect effect)

Note: * p < .05, ** p < .01, *** p < .001; NA = Not Available

Focus: IA = Internet Addiction; IBT = Impulsive Buying Tendency; IGD = Internet Gaming Disorder; PIU = Problematic Internet Usage; POG = Problematic Online Gaming; PSMU = Problematic Social Media Use; PSU = Problematic Smartphone Usage; SPA = Smartphone Addiction
EI Tool: BEIS-10 = Brief Emotional Intelligence Scale; MEIA = Multidimensional Emotional Intelligence Assessment; MSCEIT = Mayer-Salovey-Caruso Emotional Intelligence Test; SREIT = Schutte Self Report Emotional Intelligence Test; TEIQue = Trait Emotional Intelligence Questionnaire (SF = Short Form); TMMS = Trait Meta-Mood Scale; TMMS-24 = Trait Meta-Mood Scale-24 item; WLEIS = Wong and Law Emotional Intelligence Scale
Statistical Analysis: ANOVA = Analysis of Variance; CFA = Confirmatory factor analysis; EFA = Exploratory factor analysis; GLM = General linear model; MANOVA = Multivariate analysis of variance; SEM = Structural Equation Modelling; PLS-SEM = Partial least squares- structural equation modeling;

† The entries in this table highlight selected findings of interest and are not comprehensive summaries of the original research articles. For more detailed information on the study results, please refer to the respective references.
‡ Polish adaptation of the SREIT.
§ Chinese adaptation of the SREIT.
†† Despite being placed in the Ability EI section, statement of Trait EI is kept as Dang et al. (2019) did; for further explanations on this, see the discussion section.
‡‡ Although overall mean age was not reported, authors stated that 66.3% of the participants were in the age range of 16–25 (the mean age was 25.6 ± 9.19; minimum = 18, maximum = 67).

§§ This study has findings on both models of EI, therefore placed in both sections of the table (i.e., Ability EI & Trait EI)

Supplementary Table S3.

Quality Assessment of the included studies based on Adapted EPHPP (Effective Public Health Practice Project, 1998) tool

	**Study**	**Selection Bias**	**Study Design**	**Blinding**	**Data Collection Method**	**Withdrawals/ Dropouts†**	**Analyses**	**Global Rating**
**Ability EI**								
[57]	Aranda et al. (2022)	Moderate	Weak	Weak	Moderate	Not Applicable	Strong	Weak
[38]	Arrivillaga et al. (2020)	Strong	Weak	Moderate	Strong	Not Applicable	Strong	Moderate
[48]	Arrivillaga et al. (2022)	Strong	Weak	Moderate	Strong	Not Applicable	Strong	Moderate
[58]	Arrivillaga et al. (2022)	Moderate	Weak	Moderate	Strong	Strong	Strong	Moderate
[51]	Błachnio et al. (2023)	Moderate	Weak	Moderate	Weak	Not Applicable	Strong	Weak

[64]	Che et al. (2017)	Weak	Weak	Moderate	Weak	Not Applica ble	Moderate	Weak
[54]	Chen & Zhang (2023)	Moderate	Weak	Moderate	Strong	Not Applica ble	Moderate	Moderate
[60]	Dang et al. (2019)	Weak	Strong	Moderate	Strong	Weak	Moderate	Weak
[56]	Ergün & Güzel (2019)	Strong	Weak	Strong	Strong	Not Applica ble	Moderate	Moderate
[36]	Fernández-Martínez et al. (2023)	Moderate	Weak	Moderate	Weak	Not Applica ble	Moderate	Weak
[67]	Foye et al. (2019)	Moderate	Weak	Moderate	Moderate	Not Applica ble	Moderate	Moderate
[65]	Gardner et al. (2014)	Moderate	Weak	Moderate	Strong	Not Applica ble	Weak	Weak
[35]	Hoang et al. (2023)	Strong	Weak	Strong	Moderate	Not Applica ble	Strong	Moderate
[32]	Hsieh et al. (2019)	Strong	Weak	Strong	Strong	Not Applica ble	Moderate	Moderate
[49]	Jarrar et al. (2022)	Moderate	Weak	Moderate	Weak	Not Applica ble	Moderate	Weak
[71]	Jie et al. (2022)	Moderate	Weak	Moderate	Moderate	Not Applica ble	Strong	Moderate
[72]	Lekaviciene et al. (2022)	Moderate	Strong	Moderate	Weak	Strong	Moderate	Moderate
[29]	Oskenbay et al. (2015)	Weak	Moderate	Moderate	Weak	Strong	Weak	Weak
[69]	Park & Dhandra (2017)	Moderate	Weak	Moderate	Moderate	Not Applica ble	Moderate	Moderate
[30]	Saraiva et al. (2018)	Strong	Weak	Strong	Moderate	Not Applica ble	Moderate	Moderate

[59]	Torres-Rodriguez et al. (2018)	Weak	Weak	Moderate	Weak	Not Applicable	Weak	Weak
[55]	Ulwiyyah & Zhang (2022)	Moderate	Weak	Moderate	Weak	Not Applicable	Moderate	Weak
[52]	van Deursen et al. (2015)	Moderate	Weak	Moderate	Strong	Not Applicable	Strong	Moderate
[70]	Vihari et al. (2022)	Moderate	Weak	Strong	Moderate	Not Applicable	Strong	Moderate
[37]	Vu et al. (2022)	Moderate	Weak	Moderate	Moderate	Not Applicable	Strong	Moderate
[31]	Yekefallah et al. (2019)	Moderate	Weak	Weak	Weak	Not Applicable	Moderate	Weak
[34]	Yu & Zhou (2021)	Moderate	Weak	Strong	Strong	Not Applicable	Strong	Moderate
[39]	Yudes et al. (2020)	Strong	Weak	Strong	Weak	Not Applicable	Moderate	Weak
[41]	Yudes et al. (2021)	Moderate	Weak	Strong	Weak	Not Applicable	Strong	Moderate
[63]	Zahra et al. (2020)	Moderate	Weak	Weak	Moderate	Not Applicable	Weak	Weak
[40]	Zhang & Wang (2021)	Moderate	Weak	Strong	Moderate	Not Applicable	Strong	Moderate
[53]	Zhang (2023)	Strong	Weak	Strong	Strong	Not Applicable	Strong	Moderate
	**Trait EI**							
[42]	Alshakhsi et al. (2022)	Weak	Weak	Moderate	Weak	Not Applicable	Strong	Weak

[44]	Alshakhsi et al. (2023)	Weak	Weak	Moderate	Weak	Not Applicable	Strong	Weak
[66]	Andrei et al. (2018)	Moderate	Weak	Moderate	Moderate	Not Applicable	Strong	Moderate
[46]	Barberis et al. (2023)	Strong	Weak	Moderate	Strong	Not Applicable	Strong	Moderate
[68]	Biolcati et al. (2021)	Moderate	Weak	Weak	Strong	Not Applicable	Strong	Moderate
[65]	Gardner et al. (2014)	Moderate	Weak	Moderate	Weak	Not Applicable	Weak	Weak
[61]	Kırcaburun et al. (2020)	Moderate	Weak	Moderate	Moderate	Not Applicable	Strong	Moderate
[47]	Kırcaburun et al. (2019)	Moderate	Weak	Moderate	Moderate	Not Applicable	Strong	Moderate
[62]	Ko et al. (2021)	Weak	Weak	Moderate	Moderate	Not Applicable	Strong	Weak
[50]	Pino & Mastromarino (2023)	Moderate	Weak	Moderate	Weak	Not Applicable	Strong	Weak
[43]	Sechi et al. (2021)	Moderate	Weak	Moderate	Moderate	Not Applicable	Strong	Moderate
[45]	Süral et al. (2019)	Moderate	Weak	Moderate	Moderate	Not Applicable	Strong	Moderate

† Not Applicable ratings are given to the studies with cross-sectional design due to their one-time measurement method.
